# Periodicity-Driven Adaptive Localization of Respiratory Regions for Non-Contact Respiration Monitoring in Sleeping Infants

**DOI:** 10.3390/bioengineering13101158

**Published:** 2026-10-03

**Authors:** Hyoyeong Jeong, Jinsoo Kim, Eui Chul Lee

**Affiliations:** 1 Department of AI & Informatics, Graduate School, Sangmyung University, Hongjimun 2-gil 20, Jongno-gu, Seoul 03016, Republic of Korea; 202631058@sangmyung.kr (H.J.); 202635002@sangmyung.kr (J.K.); 2 Department of Human-Centered Artificial Intelligence, Graduate School, Sangmyung University, Hongjimun 2-gil 20, Jongno-gu, Seoul 03016, Republic of Korea

**Keywords:** anchor-based localization, periodicity map, grid cell, contactless infant respiration monitoring, sleep posture

## Abstract

Respiration is a critical vital sign for assessing the health status of infants during the first year of life. Conventional video-based respiratory monitoring methods often rely on manually defined regions of interest (ROIs) targeting specific anatomical regions, such as the chest or abdomen, or employ pretrained object detection models to localize body regions before extracting respiratory signals. However, in unconstrained sleep environments, infants may adopt diverse sleep postures, making it difficult to assume that respiration-induced movements will consistently appear in the same anatomical region or image location. To address this limitation, we propose an anchor-based respiratory-region localization framework that identifies spatial regions exhibiting prominent respiratory periodicity across the entire video frame without relying on predefined anatomical ROIs or separate body-region localization models. For each grid cell, time-series signals are constructed from inter-frame color/intensity variations and optical-flow-derived motion changes. The magnitude spectra of these signals are then multiplied element-wise to generate a respiratory periodicity map representing the spatial distribution of respiration-related periodicity across the frame. The grid cell with the highest Periodicity Score is selected as the Anchor Grid Cell and used as the primary respiratory region for respiratory-rate estimation. The proposed method was evaluated using 6-fold subject-wise cross-validation on 374 video clips from 15 infants. The single-Anchor Grid Cell approach achieved a mean absolute error (MAE) of 1.9953 breaths per minute (BPM), a root mean square error (RMSE) of 3.9681 BPM, and a Pearson correlation coefficient (PCC) of 0.7287. Moreover, MAEs below 2.5 BPM were obtained for the supine, side-lying, and prone postures, indicating consistent estimation performance across the evaluated sleep postures. These findings suggest that respiration-related information in infants is not necessarily confined to predefined anatomical regions and may instead be expressed at different spatial locations depending on sleep posture.

## 1. Introduction

Respiration is an important physiological measure and a critical vital sign for assessing the health status of infants during early life [1]. Infants frequently exhibit restless movements and limb activity during sleep and have more irregular respiratory patterns than adults [2,3,4]. These physiological characteristics may increase their vulnerability to serious clinical conditions, including apnea, respiratory distress, respiratory syncytial virus infection, and sudden infant death syndrome (SIDS) [1]. Notably, approximately 90% of SIDS cases occur within the first six months of life. The incidence of SIDS decreased substantially from 130.3 cases per 100,000 live births in 1990, prior to the widespread implementation of the American Academy of Pediatrics safe sleep recommendations, to 33.3 cases per 100,000 live births in 2019. Despite this decline, SIDS remains a leading cause of death among infants aged 1 month to 1 year, and the risk of SIDS-related death during the first year of life has been reported to be approximately 20 times higher than that associated with other causes [5].

Beyond the risk of SIDS, infants younger than one year are also particularly susceptible to sleep-disordered breathing, including obstructive and central sleep apnea. Their distinct anatomical characteristics and reduced muscle tone during sleep may predispose them to upper airway obstruction. In addition, central sleep apnea, characterized by a temporary cessation of airflow without accompanying respiratory effort, can occur in young infants. This vulnerability is largely attributable to the immaturity of respiratory and ventilatory control mechanisms, which may contribute to physiological instability and increased susceptibility to respiratory disturbances [4]. Consequently, there is a growing need for advanced monitoring technologies capable of continuously assessing respiratory status in sleeping infants.

At the same time, the widespread adoption of smart home devices and residential camera systems has increased interest in monitoring infant sleep in naturalistic home environments. Over the past decade, monitoring devices designed to estimate nocturnal physiological parameters, including infant respiration, have become increasingly prevalent, primarily to provide caregivers with reassurance regarding infant safety [6,7]. Conventional respiratory monitoring systems have predominantly relied on contact-based sensors. However, these systems have inherent limitations, as they may cause irritation to the delicate skin of infants, interfere with sleep due to physical discomfort, and be susceptible to motion artifacts resulting from frequent body movements. To overcome these limitations, non-contact respiratory monitoring technologies have been actively investigated [8,9].

However, owing to fundamental anatomical and physiological differences, directly applying respiratory monitoring models developed for adults to infants presents significant technical challenges [9]. Compared with adults, infants have a highly compliant chest wall and spend a substantially greater proportion of sleep time in rapid eye movement sleep. During this sleep stage, reduced respiratory muscle tone and altered respiratory mechanics may further influence their respiratory patterns [9,10]. Consequently, the spatial characteristics of respiratory movements in infants differ from those typically observed in adults. Whereas adult respiratory movements are often more prominent in the thoracic region, infants exhibit greater reliance on abdominal movement during respiration. In addition, the recruitment of accessory respiratory muscles, such as the sternocleidomastoid and scalene muscles, can produce visible shoulder movements and skin retractions around the neck [11,12,13]. As a result, infant respiratory-related displacements can occur across multiple anatomical regions rather than being confined to a single localized area [9,10]. Therefore, non-contact, vision-based respiratory monitoring systems must account for the spatial distribution of respiratory-related signals across multiple anatomical regions, including the abdomen, shoulders, and neck, rather than assuming that they are confined to the chest alone.

Moreover, in unconstrained sleep environments, infants frequently exhibit changes in sleeping posture, body rotation, and positional shifts within the camera view [14]. Such movements can lead to spatial misalignment between a Region of Interest (ROI) defined from the initial frames and the region that actually contains respiratory information. Consequently, ROIs fixed to specific anatomical locations may fail to adequately capture respiration-related signals over time [15,16]. In previous studies on non-contact respiratory monitoring, respiratory information has commonly been estimated from predefined ROIs corresponding to specific body regions, such as the face, chest, or abdomen [17,18,19]. In particular, deep learning-based vision models, including YOLO, have been employed to detect subjects and generate bounding boxes, after which ROIs are assigned to specific anatomical regions, such as the face or thoracic area. These approaches typically first detect the subject within the video, define an ROI over a predetermined body region within the detected bounding box, and subsequently estimate respiration from the signals extracted from that region [16,17,20]. Although some studies have introduced dynamic ROI strategies, these methods still fundamentally rely on object detection followed by respiration-related signal extraction from predefined regions within the detected subject area [19].

Such approaches are effective when respiratory movements are predominantly concentrated in the chest or other specific anatomical regions. However, their limitations become apparent given the physiological characteristics and variable sleep postures of infants, which can cause respiratory displacements to be distributed spatiotemporally across multiple regions of the body, including the shoulders and neck [11,12,13,21]. Therefore, reliable respiration monitoring of infants in unconstrained sleep environments requires moving beyond the conventional paradigm of first detecting a specific body region, such as the face or chest, and subsequently constraining the ROI to that location. To address this limitation, this study proposes a novel spatial respiratory-region estimation approach that identifies informative regions based on respiratory periodicity itself, rather than relying on predefined anatomical regions. Specifically, the proposed method adaptively searches the video for regions exhibiting prominent periodic displacement signals, thereby accounting for the spatial variability of respiratory movements during infant sleep. This approach is intended to facilitate robust monitoring of respiratory signals distributed across multiple regions of the body, even under unpredictable conditions involving frequent body movements and diverse sleep postures, including the prone position.

## 2. Related Work

### 2.1. Non-Contact Respiratory Estimation in Sleep Environments

Most non-contact respiratory monitoring studies have traditionally assumed adequately illuminated environments in which skin color information can be reliably captured, and have therefore employed color-based approaches that analyze subtle respiration-induced variations in skin appearance [21]. More recently, studies have explored the joint use of RGB and depth information to combine chromatic and spatial cues, with the aim of maintaining reliable respiratory signal estimation even under low-light conditions [8]. In a related study, the color-based CHROM algorithm was applied to videos of spontaneous respiration and demonstrated strong agreement with electrocardiography (ECG), achieving correlation coefficients greater than 0.86 not only under visible-light conditions but also in nighttime infrared (IR) settings within an incubator, indicating stable respiratory rate estimation across different illumination conditions [22].

In parallel with color-based approaches, optical flow-based methods have been investigated to capture subtle spatial movements associated with respiration by estimating inter-frame motion. Several studies have employed the resulting motion vectors as inputs to spatiotemporal deep learning models for respiratory signal estimation [1,16]. Other approaches have combined optical flow with thermal imaging to jointly analyze respiratory motion and temperature variations around the nasal region [17]. Optical flow is particularly useful for non-contact respiratory monitoring because it enables respiration-related motion patterns to be characterized even when reliable color information is unavailable or limited. A comparative study using infant sleep videos further reported that not only conventional optical flow methods, such as DeepFlow, but also deep learning-based physiological signal estimation models, such as EfficientPhys, could achieve stable performance under specific imaging conditions [16].

### 2.2. Limitations of ROI Extraction in Existing Respiratory Monitoring

To identify the ROI containing respiratory information, a top-down approach has been widely adopted; this method first detects the target subject using deep learning-based object detection models such as YOLO, Faster R-CNN, and DINO and subsequently extracts the ROI [16,19,23,24]. This procedure typically involves detecting specific anatomical regions (e.g., the face, chest, and abdomen) using weights pre-trained on large-scale adult RGB datasets, such as MS COCO, and establishing these areas as the ROI [17,18]. Within the single region extracted via this methodology, traditional machine learning techniques have been applied to track the physical displacement of pixels, or these displacements have been utilized as inputs for deep learning models to estimate the respiratory signal [25]. However, this paradigm predicated on preliminary object detection and fixed ROIs exhibits distinct limitations when applied to infant respiratory monitoring. Fixed ROI techniques that rely on a single, specific region, such as the chest or body, fail to adequately capture the complex and diverse respiratory mechanisms unique to infants. Consequently, this leads to a degradation in the signal-to-noise ratio and overall respiratory prediction performance [16].

Recently, related studies have proposed dynamic ROI extraction methods to address the limitations associated with fixed ROIs. Various methodologies have been reported that dynamically search for regions containing respiratory signals by comprehensively utilizing RGB-D (depth) information, thermal imaging, optical flow-based motion analysis, or convolutional neural network-based feature extraction and object detection models [14,19,26]. Nevertheless, these methods still fundamentally adopt a pipeline in which the target subject is defined initially, and respiratory information is estimated strictly within the corresponding bounding box [19]. Therefore, because the analytical scope of the respiratory information is dictated by the initial object detection results, these approaches suffer from a fundamental limitation wherein the overall respiratory estimation performance is heavily bottlenecked by the accuracy of the initial object detection phase.

## 3. Method

This study proposes a framework that adaptively identifies valid respiratory regions across entire IR and RGB sleep videos without relying on manually defined ROIs or pretrained object detection models for detecting specific body regions. The proposed framework aims to identify and analyze regions in which respiration-related information is manifested across different sleep postures. Each video frame is divided into a grid, and time-series signals are constructed for each grid cell based on temporal color/intensity variations and optical flow-derived motion changes. The periodicity of these signals is then analyzed to assess respiratory periodicity for each grid cell, and the grid cell exhibiting the highest respiratory periodicity is selected as the valid respiratory region. Finally, the signal from the selected grid cell is used to estimate the respiratory rate for the corresponding video.

### 3.1. Dataset

From the AIR-400 dataset [16], the initially collected YouTube-based videos from AIR-125 [1] were excluded from the present analysis because the links to the original videos could not be verified. The dataset consists of natural sleep videos recorded during the day and night in home environments and includes various sleep postures and spontaneous movements in infants aged 4–11 months. The final dataset comprised 374 video clips from 15 infants, including 50 RGB videos and 324 IR videos. With respect to sleep posture, 29 videos were recorded in the prone position, 120 in the side-lying position, and 225 in the supine position. All videos were exactly 1 min in duration, with frame rates of either 10 or 15 frames per second (FPS). The actual FPS of each video was determined individually and used accordingly in the subsequent analysis. The ground-truth respiratory waveform was constructed based on peak timestamps annotated with reference to the onset of exhalation, corresponding to the transition point from inhalation to exhalation.

The preprocessing pipeline primarily consists of two steps. First, to reduce the influence of the recording timestamp displayed at the top of the original video during color-difference computation, the uppermost 10% of each frame was cropped. Second, to reduce the computational cost of Farnebäck optical flow estimation, the resulting cropped frames were subsequently resized to 20% of their dimensions.

**Figure 1:**
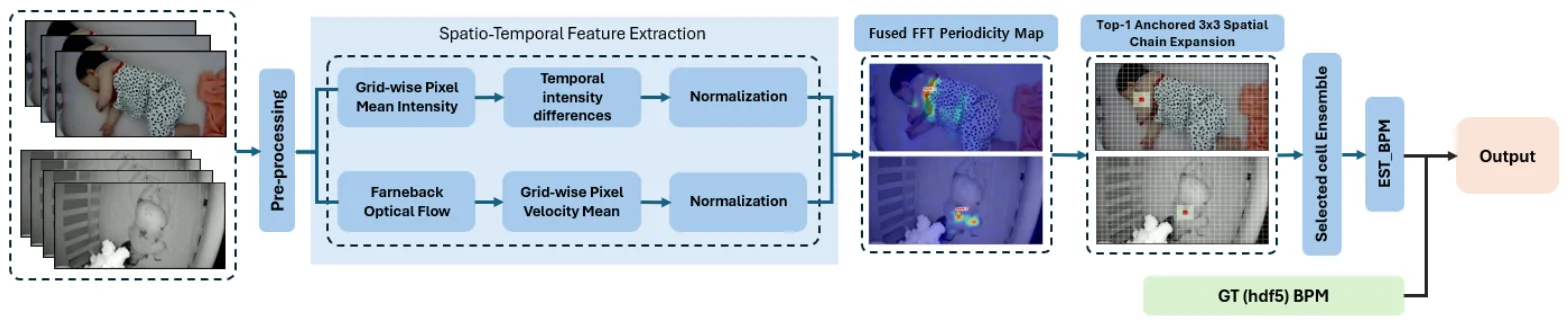
Adaptive respiratory region localization pipeline. Note. The proposed framework adaptively identifies respiration-related regions in RGB and IR sleep videos by analyzing temporal color/intensity variations and optical flow-derived motion changes to detect regions exhibiting respiratory periodicity. The input consists of 1-min RGB or IR video clips, from which two features, Color Difference and Optical Flow, are extracted during the Spatio-Temporal Feature Extraction stage. FFT is then applied separately to the two extracted features, and a Fused FFT Periodicity Map is generated by multiplying the resulting frequency spectra. Respiratory periodicity is subsequently evaluated for each Grid Cell based on the Fused FFT Periodicity Map, and the Grid Cell exhibiting the highest periodicity is selected as the Anchor Grid Cell (Top-1). The selected Anchor Grid Cell serves as the primary respiration-related region and is used for respiratory rate estimation. As an optional extension, neighboring Grid Cells satisfying the predefined correlation criterion can additionally be incorporated through Chain Extension.

Following preprocessing, the spatio-temporal feature extraction stage performs two complementary operations, color differences and optical flow, regardless of the input modality (RGB or IR). Color difference is computed using the *C_r_* − *C_b_* channel difference for RGB images, whereas the temporal difference in grayscale intensity is used for IR images. For optical flow computation, RGB images are first converted to grayscale, whereas the original IR images are directly used as input (Figure 1).

### 3.2. Grid-Based Temporal Color/Intensity Difference Computation

(1) Grid-wise Pixel Mean Intensity

The preprocessed frames of the input video are partitioned into uniform grids, and a representative signal is subsequently generated for the pixels within each grid cell according to the image mode (RGB or IR). For RGB images, the difference signal between the red-difference (*C_r_*) and blue-difference (*C_b_*) components in the YCrCb color space is utilized to ensure robustness against illumination changes and to capture subtle variations in microvascular blood flow and skin tone. In contrast, for IR images, the intensity value (*I*_gray_) is used, as it is a single-channel grayscale image. The spatial average signal *S*_grid_(*y*,*x*,*t*) is calculated as shown in Equation (1).

(1) $$S_{\mathrm{grid}}\left(y,x,t\right)=\left\{\begin{array}{cc} \frac{1}{H_{g}W_{g}}\sum_{i=0}^{H_{g}-1}\sum_{j=0}^{W_{g}-1}\left(C_{r}\left(i+y_{\mathrm{start}},j+x_{\mathrm{start}},t\right)-C_{b}\left(i+y_{\mathrm{start}},j+x_{\mathrm{start}},t\right)\right), & \mathrm{if RGB Video}\\ \frac{1}{H_{g}W_{g}}\sum_{i=0}^{H_{g}-1}\sum_{j=0}^{W_{g}-1}I_{\mathrm{gray}}\left(i+y_{\mathrm{start}},j+x_{\mathrm{start}},t\right), & \mathrm{if IR Video} \end{array}\right.$$

Here, *H_g_* and *W_g_* represent the height and width of the grid cell, respectively, and *y*_start_ = *g_y_H_g_* and *x*_start_ = *g_x_W_g_* denote the starting coordinates of the corresponding grid cell. *C_r_*(*y*,*x*,*t*) and *C_b_*(*y*,*x*,*t*) are the red-difference and blue-difference channel values of the RGB frame converted into the YCrCb color space, whereas *I*_gray_(*y*,*x*,*t*) represents the intensity channel value of the IR frame.

(2) Temporal Differences & Normalization

The mean intensity difference between two adjacent frames is calculated. Subsequently, to reduce the signal scale differences between grid cells, Z-score normalization is performed using the mean and standard deviation (SD). The difference and normalization processes are defined by Equations (2) and (3).

Δ*S*(*y*,*x*,*t*) = *S*_grid_(*y*,*x*,*t*) − *S*_grid_(*y*,*x*,*t* − 1) (2)

(3) $$C_{\mathrm{norm}}\left(y,x,t\right)=\frac{\Delta S\left(y,x,t\right)-\mu_{C}\left(y,x\right)}{\sigma_{C}\left(y,x\right)+\epsilon }\;\left(\epsilon =10^{-8}\right)$$

### 3.3. Grid-Based Optical Flow Computation

(1) Farnebäck Optical Flow

To capture respiratory movements within the video, Gunnar Farnebäck’s dense optical flow algorithm was applied [27]. This method approximates local neighborhoods in two consecutive frames using quadratic polynomials to estimate pixel displacement vectors, thereby providing dense motion information across the video. The computation is performed using the preprocessed frames as shown in the following Equation (4).

(4) $$\begin{array}{c} V\left(y,x,t\right)=\mathrm{FarnebackFlow}{\left(I_{t-1},I_{t}\right)}_{y,\mathrm{component}} \end{array}$$

where *V*(*y*,*x*,*t*) represents the optical flow velocity component at spatial coordinates y, x and time t, extracted via the Farnebäck optical flow algorithm between consecutive frames *I_t_*_−1_ and *I_t_*.

(2) Grid-wise Pixel Velocity Mean & Normalization

For each grid cell, the velocity components of the enclosed pixels are averaged to obtain a representative motion signal. Z-score normalization is then applied to reduce differences in signal scale across grids, as defined in Equation (5).

(5) $$F_{\mathrm{norm}}\left(y,x,t\right)=\frac{V\left(y,x,t\right)-\mu_{F}}{\sigma_{F}+\epsilon }$$

where *μ_F_* and *σ_F_* denote the temporal mean and SD, respectively, of the optical flow signal *V*(*y*, *x*, *t*) for the grid cell at (*y*, *x*), and *ϵ* = 10^−8^ is a small constant introduced for numerical stability to prevent division-by-zero errors.

### 3.4. FFT-Based Fused Periodicity Map

The frequency spectrum is calculated by performing Fast Fourier Transform (FFT) on the normalized intensity variation signal and the optical flow signal, respectively. Subsequently, the two spectra are multiplied in the frequency domain to create a map that emphasizes the periodic components where color variations and motion variations occur simultaneously. This process is defined in Equation (6).

(6) $$\begin{array}{c} Y_{c}\left(f\right)=\left|\mathrm{FFT}\left(C_{\mathrm{norm}}\left(y,x,t\right)-\mu_{s}\right)\right|\\ Y_{f}\left(f\right)=\left|\mathrm{FFT}\left(F_{\mathrm{norm}}\left(y,x,t\right)-\mu_{s}\right)\right|\\ Y_{\mathrm{fused}}\left(f\right)=Y_{c}\left(f\right)\cdot Y_{f}\left(f\right) \end{array}$$

where *C_norm_*(*y*,*x*,*t*) and *F_norm_*(*y*,*x*,*t*) denote the normalized intensity variation signal and the optical flow signal, respectively, and *μ_s_* is the mean value for removing the temporal axis mean in the corresponding grid cell. By multiplying the two spectra in the frequency domain, the noise components that may occur in a single signal are reduced, and the respiration-related periodic components that appear simultaneously in both color and motion variations are selectively enhanced.

Within the generated Periodicity Map, the periodicity of each grid cell is quantified using a Periodicity Score, defined as the ratio of the maximum fused FFT magnitude within the respiratory frequency band (0.2–0.8 Hz) to the sum of the fused FFT magnitudes over the entire spectrum, as shown in Equation (7).

(7) $$\begin{array}{c} \mathrm{Score}\left(y,x\right)=\frac{\underset{f\in \left[0.2,0.8\right]}{\max}Y_{\mathrm{fused}}\left(f\right)}{\sum_{f}Y_{\mathrm{fused}}\left(f\right)+\epsilon } \end{array}$$

where *ϵ* is a small constant to prevent the denominator from becoming zero. Using the calculated Periodicity Score, all grid cells are sorted in descending order, and the grid cell with the highest score is selected. Herein, the selected grid cell is referred to as the Anchor Grid Cell.

### 3.5. Correlation-Based Spatial Chain Extension

In addition to the primary single-Anchor Grid Cell approach, we experimentally evaluated a correlation-based Chain Extension strategy to examine whether incorporating spatially adjacent grid cells provides additional benefit for respiratory rate estimation. Starting from the Anchor Grid Cell, the Pearson correlation coefficient was calculated between its respiratory time-series signal and those of candidate cells within a 3 × 3 neighborhood. Neighboring candidate cells satisfying a correlation threshold of ρ ≥ 0.9 were incorporated into the extended respiratory region and designated as new Anchor Grid Cells. Using each newly designated Anchor Grid Cell as an anchor, the same 3 × 3 neighborhood search was recursively performed, and additional cells satisfying the correlation criterion were incorporated. This iterative procedure spatially expands the connected respiratory region while retaining neighboring cells that exhibit highly correlated temporal patterns with the Anchor Grid Cell. The correlation-based Chain Extension procedure is formally defined in Equation (8).

(8) $$S_{\mathrm{fused}}=W_{\mathrm{flow}}\cdot S_{\mathrm{flow}}^{\mathrm{norm}}+W_{\mathrm{color}}\cdot S_{\mathrm{color}}^{\mathrm{norm}}$$

*s*_1_(*t*) = *S*_fused_(*c*_anchor_,*t*), *s*_2_(*t*) = *S*_fused_(*c*_target_,*t*)

where *s*_1_(*t*) and *s*_2_(*t*) represent the dual-modality weighted fused time-series signals extracted from the anchor cell and the adjacent candidate cells within the 3 × 3 spatial range at frame *t*, respectively. *W*_flow_ and *W*_color_ are the weights applied to the optical flow and color signals, respectively, and $S_{\mathrm{flow}}^{\mathrm{norm}}$ and $S_{\mathrm{color}}^{\mathrm{norm}}$ denote the normalized time-series signals extracted from each modality. Through this, the respiration-related information of the two modalities is configured into a single fused time-series signal.

Subsequently, to remove the baseline offset, the mean *μ*(*s*) was subtracted from each signal, yielding the zero-centered signals *s*_1_′ and *s*_2_′. The term *ϵ* in the denominator serves as a numerical stabilization term to prevent division-by-zero errors when the signal variance is extremely small. This operation is defined in Equation (9).

(9) $$\begin{array}{c} {s_{1}}^{\prime }=s_{1}-\mu \left(s_{1}\right),\;{s_{2}}^{\prime }=s_{2}-\mu \left(s_{2}\right)\\ \rho \left(s_{1},s_{2}\right)=\frac{\sum_{t}\left({s_{1}}^{\prime }\left(t\right)\cdot {s_{2}}^{\prime }\left(t\right)\right)}{\sqrt{\sum_{t}{\left({s_{1}}^{\prime }\left(t\right)\right)}^{2}\sum_{t}{\left({s_{2}}^{\prime }\left(t\right)\right)}^{2}}+\epsilon } \end{array}$$

### 3.6. Respiration Rate Estimation

For the primary configuration of the proposed framework, the respiratory signal is obtained from the single Anchor Grid Cell exhibiting the highest Periodicity Score. The signal is subsequently band-pass filtered within the infant respiratory frequency range of 0.2–0.8 Hz using a Butterworth filter. Fast Fourier Transform (FFT) is then applied to calculate the frequency spectrum, and the frequency exhibiting the maximum amplitude within the respiratory band is selected as the dominant respiratory frequency. This frequency is converted into breaths per minute (BPM) to obtain the estimated respiratory rate.

For the optional Chain Extension configuration described in Section 3.5, the time-series signals of the Anchor Grid Cell and the additionally selected neighboring cells are first averaged to obtain an aggregated respiratory signal. The same Butterworth band-pass filtering and FFT-based respiratory rate estimation procedure is then applied to this aggregated signal.

(10) $$\begin{array}{c} f_{k}=\frac{k}{N\cdot \Delta t}=\frac{k\cdot f_{s}}{N}\\ f_{\mathrm{peak}}=\arg\underset{f_{k}\in \left[0.2,0.8\right]}{\max}\left|Y\left(f_{k}\right)\right|\\ \mathrm{est}\text{\_}\mathrm{bpm}=f_{\mathrm{peak}}\times 60.0 \end{array}$$

where *N* represents the total number of frames, and *f_s_* represents the sampling frequency, where *f_k_* denotes the discrete frequency corresponding to the bin index *k*. This process is defined in Equation (10).

## 4. Results

**Figure 2:**
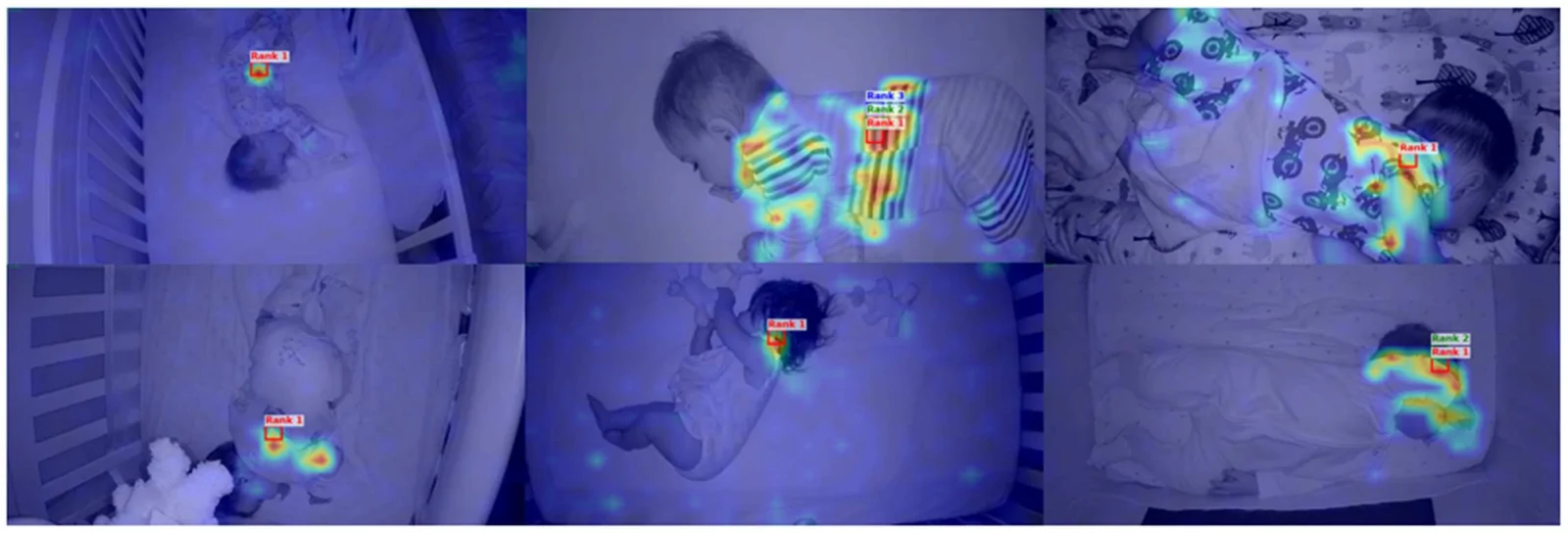
Spatial distribution of respiratory periodicity across grid cells. Note. The heatmaps visualize the spatial distribution of respiratory periodicity across grid cells and highlight the regions estimated to contain respiration-related information. Using the Anchor Grid Cell (Rank 1), which corresponds to the highest Periodicity Score, as a reference, adjacent grid cells within a 3 × 3 region that satisfy the Pearson correlation coefficient threshold (r ≥ 0.9) are selected and included in the chain extension. The red shades in the heatmap indicate the regions where the respiratory periodicity is most strongly exhibited, whereas the blue shades represent the regions where it is the weakest.

To verify the spatial locations of the estimated respiration-related regions, the generated periodicity map was visualized as a heatmap overlaid on the corresponding video frames. By analyzing the periodic characteristics of the time-series signals extracted from each grid cell over the entire video, the grid cell containing the strongest respiration-related information was adaptively selected as the anchor according to the infant’s sleep posture. Regions exhibiting high respiratory periodicity are represented in red, indicating that prominent respiration-related information can be localized to different body regions, including the abdomen, shoulders, neck, and back, depending on the infant’s sleep posture. In contrast, regions exhibiting low respiratory periodicity are represented in blue, indicating a clear distinction from regions containing stronger respiration-related periodic information (Figure 2).

The experimental results showed that the use of a single Anchor Grid Cell yielded slightly lower MAE and RMSE values and a slightly higher PCC than the aggregation of multiple Grid Cells. Although the overall performance differences were small, the single Anchor Grid Cell consistently achieved the best performance among the evaluated configurations, with an MAE of 1.9953. These findings suggest that accurately localizing the region containing the most prominent respiration-related information may be more important than simply aggregating information from a larger number of Grid Cells. In addition, the use of a single Anchor Grid Cell may limit the inclusion of signals from surrounding cells with weaker respiratory periodicity or non-respiratory motion, thereby reducing the potential contribution of noise to the estimated respiratory signal (Table 1).

As shown in Table 2, the Chest and Body ROIs yielded MAEs of 4.1893 and 4.0394 BPM and RMSEs of 6.8649 and 6.8765 BPM, respectively, with corresponding PCC values of 0.4094 and 0.4111. The Torso ROI showed an MAE of 6.0633 BPM, an RMSE of 9.0863 BPM, and a PCC of −0.0194. In comparison, the proposed Anchor Grid Cell approach achieved an MAE of 2.1836 BPM, an RMSE of 4.2289 BPM, and a PCC of 0.7216. Thus, within the common evaluation subset, the Anchor Grid Cell approach exhibited lower estimation errors and a higher correlation with the reference respiratory rates than the predefined anatomical ROI strategies. All ROI strategies were evaluated using the same subset of 327 videos to maintain a consistent comparison. Accordingly, the Anchor Grid Cell performance reported in Table 2 represents the result for this N = 327 subset and differs from the primary performance result obtained using the complete 374-video dataset.

Posture-specific analysis showed that respiration rate estimation performance varied across the three evaluated sleep postures. For the supine posture, the MAE and RMSE were 1.9823 and 4.1492 BPM, respectively, with a PCC of 0.5252. The side-lying posture exhibited an MAE of 2.1508 BPM and an RMSE of 3.9290 BPM, while showing the highest PCC among the three postures (0.8423). In contrast, the prone posture showed the lowest estimation errors, with an MAE of 1.4531 BPM and an RMSE of 2.3697 BPM, but had the lowest PCC (0.3071). Overall, the MAE remained below 2.5 BPM across all three postures, indicating relatively consistent error performance across the evaluated sleep postures (Table 3).

Subsequently, the proposed framework primarily estimates the respiratory signal centered on the Anchor Grid Cell. To determine the optimal correlation threshold for the Chain Extension and the optimal fusion ratio between the two modalities (Flow and Color), a parameter grid search was conducted using the training data only. As shown in Table 4, Avg_Selected_Cells exhibited a consistent trend with respect to the threshold, regardless of the fusion weight ratio. As the threshold increased from 0.5 to 0.9, Avg_Selected_Cells decreased from 6.59 to 1.81. This indicates that, within the 3 × 3 region, an average of 6.59 of the eight neighboring cells were selected even under the most relaxed condition (threshold = 0.5). These results demonstrate that the number of neighboring cells included in the Chain Extension is controlled by the correlation threshold. Accordingly, a higher threshold imposes a stricter selection criterion, resulting in the inclusion of only neighboring cells exhibiting stronger correlation with the Anchor Grid Cell.

A consistent improvement in estimation performance was also observed across all threshold intervals, with MAE decreasing and PCC increasing as the optical flow weight increased and the color-difference weight decreased. Based on these results, the optimal performance was achieved at a threshold of 0.9 with an optical flow weight of 0.9 and a color-difference weight of 0.1, yielding the lowest MAE and Root Mean Square Error (RMSE) (2.0119 and 4.1216 BPM, respectively) and the highest PCC and ICC (0.7332 and 0.7251, respectively). Subsequent spatial search analysis further showed that expanding the search range from 3 × 3 (Avg_Selected_Cells: 1.81, MAE: 2.0119 BPM, PCC: 0.7332) to 5 × 5 (Avg_Selected_Cells: 1.86, MAE: 2.0108 BPM, PCC: 0.7339) resulted in nearly identical estimation performance, with only marginal changes in MAE and PCC.

The deep learning-based methods, EfficientPhys and AIRFlowNet, achieved MAEs of 4.034 BPM and 3.926 BPM, respectively, with corresponding PCC values of 0.377 and 0.445, demonstrating overall performance comparable to that of the Optical Flow- and Color Differences-based methods. In contrast, the Anchor Grid Cell-based approach achieved an MAE of 1.9953 BPM and a PCC of 0.7287, yielding the best overall performance among the evaluated methods. The Anchor Grid Cell + Chain Extension approach, which additionally incorporated neighboring regions, achieved an MAE of 2.1510 BPM and a PCC of 0.6714 (Table 5).

**Figure 3:**
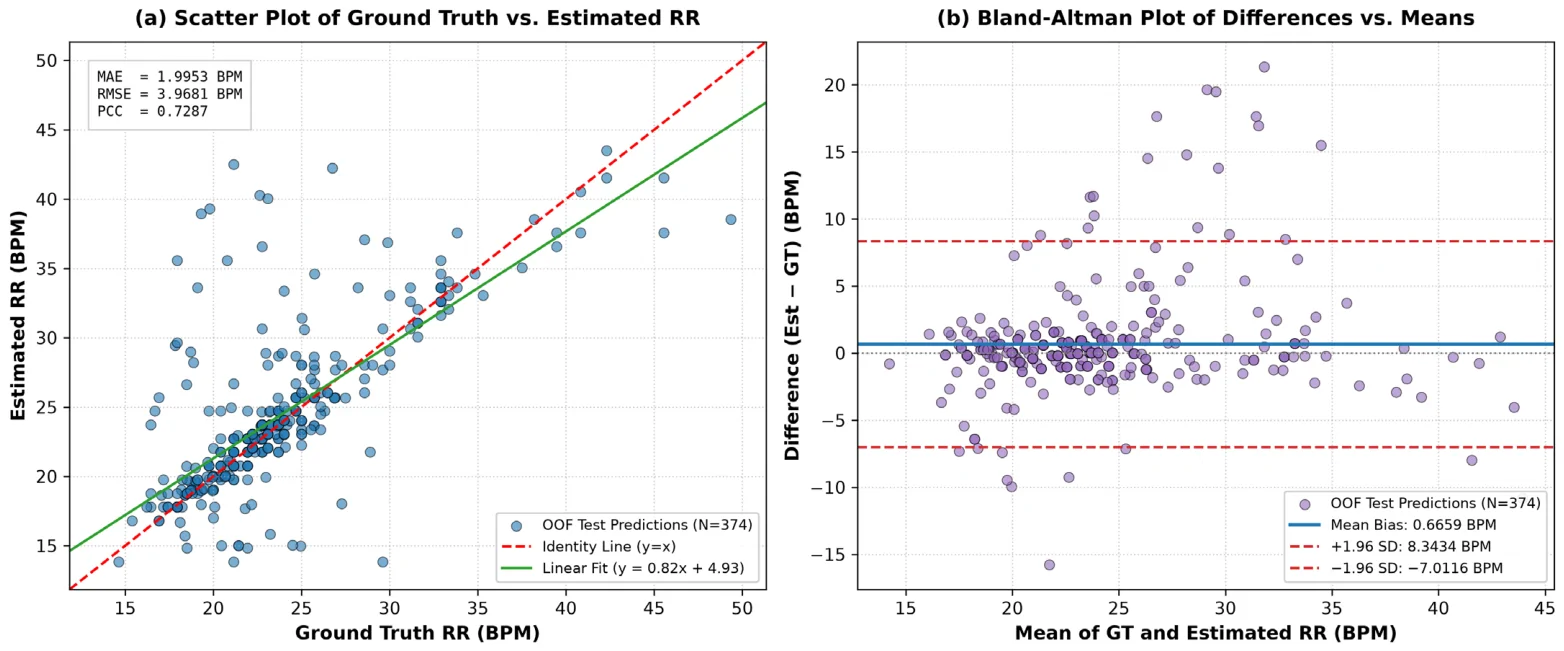
Out-of-fold (OOF) prediction results based on 6-fold subject-wise cross-validation. Note. The left image (**a**) illustrates the relationship between the ground-truth (GT) respiration rate and the estimated respiration rate across the dataset. The red dashed line represents the ideal line of identity, and a tighter clustering of points around this diagonal dashed line indicates a lower estimation error. The right image (**b**) presents the Bland–Altman plot, displaying the difference between the estimated respiration rate and GT respiration rate as a function of their average. The solid red line indicates the mean bias (0.6659 BPM), and the dashed gray lines represent the 95% limits of agreement (mean bias ± 1.96 SD). CV denotes cross-validation.

Accordingly, the single Anchor Grid Cell, which yielded the best performance, was selected as the final signal source, and a 6-fold subject-wise cross-validation (CV) was conducted based on this configuration. As shown in the results on the left side of Figure 3a, the proposed method achieved an MAE of 1.9953 BPM and a PCC of 0.7287, with the estimated respiratory rates generally distributed around the ideal agreement line. In addition, the Bland–Altman analysis on the right side of Figure 3 was conducted to assess the agreement between the ground-truth (GT) respiratory rates and the estimates produced by the proposed algorithm. The mean bias was +0.6659 BPM, indicating minimal systematic bias. The 95% limits of agreement ranged from −7.01 to +8.34 BPM. Examination of the data distribution further revealed that, in the relatively stable range of respiratory rates below 25 BPM, the estimation errors were concentrated near zero, indicating high agreement with the GT values. In contrast, at higher respiratory rates (>25 BPM), the error distribution became more dispersed, and several outliers were observed in which the respiratory rate was overestimated relative to the GT values (Figure 3).

The quantitative results in Table 6 show that an optical flow-to-color difference fusion weight ratio of 0.9:0.1 was consistently selected across all folds. Among the individual folds, Fold 6 achieved the lowest estimation errors, with an MAE of 1.2616 BPM and an RMSE of 2.2819 BPM, while also attaining a PCC of 0.8525. In contrast, Fold 4 yielded a slightly higher MAE of 1.3188 BPM, approximately 0.06 BPM higher than that of Fold 6, but achieved the highest linear correlation and agreement, with a PCC of 0.9615 and an ICC of 0.9562. Meanwhile, Fold 5 exhibited the lowest overall correlation and agreement, with an MAE of 2.8989 BPM, a PCC of 0.1321, and an ICC of 0.1075. These results indicate substantial variability in correlation-based performance across folds despite the consistent selection of the same fusion weight ratio.

When the performance was compared by varying only the combination strategy of the two signals, Spectral Multiplication corresponded to the same combination approach used in the proposed method. The Weighted Average method achieved an MAE of 2.3496 BPM and a PCC of 0.6266, corresponding to an approximately 0.4 BPM higher MAE and a 0.1 lower PCC than those of the proposed method. The Optical Flow-only method showed slightly lower performance than the Weighted Average method, whereas the Color-Intensity Differences-only method exhibited the highest MAE and the lowest PCC among the evaluated approaches. These results indicate that respiratory rate estimation performance varied according to the signal combination strategy under otherwise identical signal-processing conditions (Table 7).

To evaluate robustness to non-respiratory periodic motion, a controlled interference analysis was performed by introducing a 0.6-Hz sinusoidal disturbance within the respiratory frequency band. As shown in Table 8, respiratory-rate estimation performance progressively deteriorated with increasing disturbance amplitude under both global and localized contamination. Across all evaluated amplitudes, the MAE was lower under localized contamination than under global contamination. In addition, the proportion of anchors relocated outside the contaminated region increased from 5.1% at an amplitude of 0.1 to 48.9% at an amplitude of 0.5.

## 5. Discussion

In this study, comparison of the weighting combinations of Color Differences and Optical Flow across the evaluated threshold range revealed a consistent trend: increasing the relative weight of Optical Flow was associated with lower MAE and RMSE and higher PCC values (Table 4). This pattern may be related to the characteristics of real-world home sleep environments, in which infants are frequently partially covered by clothing, blankets, or other bedding materials. Under such conditions, the amount of informative respiratory signal available to the Color Differences-based approach, which relies on temporal variations in image intensity and color, may be limited when the infant’s skin surface is partially obscured. In contrast, Optical Flow does not directly depend on visible skin appearance but instead captures spatiotemporal variations in motion within the video. It may therefore be relatively more effective in reflecting subtle respiration-related body movements even when skin exposure is restricted by clothing or bedding [28]. Consistent with this interpretation, previous research has reported that DeepRespNet achieved respiratory estimation across different skin tones by combining hemodynamic changes in the facial region with optical-flow displacement observed in the upper chest region [29].

In addition to these differences in the relative contributions of the two signals, the ablation study results presented in Table 7 showed that using either optical flow or color-intensity differences alone resulted in slightly lower performance than using both modalities together. This finding suggests that the two signals may provide complementary respiratory information. Optical flow can capture subtle motion associated with respiration, although it may also be affected by non-respiratory body movements. In contrast, color-intensity differences can reflect local variations in color and brightness associated with respiration but may be relatively more sensitive to changes in illumination conditions and image contrast. Therefore, combining the two modalities may partially compensate for the limitations of each individual signal and provide a more stable representation of respiration-related periodic information.

To further evaluate the effect of non-respiratory periodic motion, a controlled interference analysis was performed by introducing a 0.6-Hz sinusoidal disturbance within the respiratory frequency band. As shown in Table 8, respiratory-rate estimation performance progressively deteriorated with increasing disturbance amplitude under both global and localized contamination. Across all evaluated amplitudes, the MAE was lower under localized contamination than under global contamination, while the proportion of anchors relocated outside the contaminated region increased as the disturbance amplitude increased. These findings suggest that the periodicity-driven spatial search can partially avoid spatially localized interference by relocating the Anchor Grid Cell to an alternative region exhibiting stronger respiratory periodicity. In contrast, when periodic interference was distributed globally across the frame, the benefit of spatial re-localization became limited because alternative uncontaminated regions were no longer available. Therefore, the proposed approach provides a degree of robustness to spatially localized periodic interference, whereas sufficiently strong or widespread motion within the respiratory frequency range can still degrade respiratory-rate estimation. Because this analysis was based on synthetic single-frequency interference, further validation using naturally occurring gross body movements is warranted.

As an optional extension to the primary Single Anchor Grid Cell strategy, Chain Extension was evaluated to determine whether spatially correlated neighboring regions could provide additional respiratory information. In this configuration, the search was expanded from the Anchor Grid Cell with the highest periodicity score to neighboring cells within a 3 × 3 region, and neighboring cells were included according to the correlation between their respiratory signals and that of the Anchor Grid Cell. A correlation threshold (τ) was therefore introduced as the selection criterion for this optional spatial extension. As shown in Table 4, increasing the threshold from 0.5 to 0.9 was associated with lower MAE and RMSE and higher PCC values, suggesting that stricter correlation-based selection may reduce the inclusion of neighboring cells with weaker respiratory relevance. However, expanding the search range from 3 × 3 to 5 × 5 resulted in only minimal changes in estimation performance, indicating that the selection criterion for neighboring cells may be more influential than the spatial extent of the search region itself.

Despite this correlation-based selection, Chain Extension did not improve respiratory rate estimation compared with the Single Anchor Grid Cell configuration. As shown in Table 5, the Single Anchor Grid Cell achieved an MAE of 1.9953 BPM and a PCC of 0.7287, whereas the Chain Extension configuration yielded an MAE of 2.1510 BPM and a PCC of 0.6714. This result suggests that the grid cell with the strongest respiratory periodicity may already contain sufficiently informative respiratory signals under the evaluated conditions. Incorporating additional neighboring cells may introduce weaker respiratory components or unrelated local motion, thereby reducing the quality of the aggregated signal. Accordingly, the Single Anchor Grid Cell was retained as the primary configuration of the proposed framework, whereas Chain Extension was treated as an optional spatial refinement evaluated through ablation analysis. Although such an extension could potentially be beneficial when respiratory information within a single region is locally degraded, this possibility was not directly examined in the present study and warrants further investigation.

The primary objective of the present study was not to determine the relative superiority of a particular anatomical ROI, but to adaptively localize valid respiration-related regions while accounting for the possibility that such information may appear at different spatial locations depending on posture and imaging conditions. In the ROI comparison, predefined Chest, Body, and Torso regions were obtained using the pretrained YOLOv8 model provided for the dataset and were evaluated using the same downstream respiratory signal extraction and estimation procedures. From this perspective, the proposed Anchor Grid Cell approach differs from predefined anatomical ROI strategies in that it does not require selection of a specific body region in advance, but instead identifies the spatial region exhibiting the strongest respiratory periodicity directly from the image.

Previous studies have reported that respiratory-induced contraction and expansion of the thoracic cavity can be mechanically transmitted to adjacent body regions, producing respiration-synchronized movements even in areas without direct thoracic motion [30]. This suggests that, even when the chest is partially occluded by the infant’s body or external objects, respiration-related periodic movements may still be observable in adjacent regions such as the arms, shoulders, or head. In a related study, multiple ROIs, including the forehead, cheeks, and both shoulders, were defined using YOLO, and respiratory signals were estimated from each region [23]. These observations indicate that respiratory information is not necessarily confined to a single anatomical region and may be distributed across multiple body surface locations. This provides further rationale for adaptively identifying regions that exhibit prominent respiration-related periodicity, rather than constraining the analysis to a predefined anatomical location.

In addition, the quantitative comparison across sleep postures showed that the prone posture achieved the lowest MAE of 1.4531 BPM. However, given the relatively small proportion of prone-posture videos (7.80%), this result alone is insufficient to characterize respiratory estimation performance in the prone posture more broadly. Interpretation of the prone-posture results should therefore consider not only the quantitative performance metrics but also the spatial distribution of respiratory information and the anatomical characteristics of this posture (Table 3). In particular, because the chest and abdomen are in contact with the underlying surface in the prone posture, direct thoracoabdominal movements may be less clearly observable in the video than in the supine posture. Instead, respiration-induced motion may be manifested indirectly through the back or surrounding body regions. Moreover, changes in muscle tone and body posture during sleep may alter how respiration-related movements are transmitted to the body surface depending on the regions in contact with the underlying surface. These characteristics may limit the applicability of conventional chest- and abdomen-based ROI approaches that primarily assume direct observation of thoracoabdominal movements across different infant sleep postures [9,10].

To further examine the feasibility of continuously updating respiratory-rate estimates, a supplementary streaming-style analysis was conducted using 51 approximately 5-min continuous recordings. Following initial Anchor Grid Cell localization using the first 60 s of each recording, respiratory-rate estimates were updated every 1 s using a 60-s sliding window. The anchor was retained by default and was reassessed only when predefined signal-instability criteria were satisfied, rather than being globally re-selected at every update. The implementation was executed using CPU-based processing without GPU acceleration and achieved a mean processing throughput of 45.00 ± 2.97 frames/s and a mean compute real-time factor (RTF) of 0.234 ± 0.032. An RTF below 1 indicates that the computational processing time was shorter than the corresponding video duration, indicating computational feasibility for continuous processing. Nevertheless, because this analysis was performed on prerecorded videos rather than in an online acquisition environment, these results should be interpreted as evidence of computational feasibility for real-time processing rather than as formal validation of a real-time monitoring system.

As shown in Table 6, Fold 4 exhibited a high linear correlation (PCC = 0.9615), corresponding to a wide distribution of GT respiratory rates (GT range: 19.74–49.34 BPM; SD: 7.97 BPM). In contrast, the GT respiratory rates in Fold 5 were concentrated within a relatively narrow range of 18.71–29.87 BPM (GT span: 11.16 BPM; SD: 2.43 BPM), resulting in a substantially lower PCC of 0.1321. This reduced correlation may be partly attributable to the range-restriction effect, whereby limited variability in the GT values constrains the observable linear correlation between the estimated and reference values. Nevertheless, Fold 5 maintained a relatively low estimation error, with an MAE of 2.8989 BPM, indicating that the low PCC does not necessarily imply poor absolute estimation accuracy and may instead be influenced by the restricted variability of the GT respiratory rates. To further examine this relationship, a correlation analysis was performed between the GT respiratory rate span and the PCC values across all six folds. The analysis yielded a relatively strong positive correlation (r = 0.7915), indicating that folds with wider GT respiratory rate ranges tended to exhibit higher PCC values. This result suggests that GT variability may substantially influence the observed PCC and should therefore be considered when interpreting fold-level correlation performance.

## 6. Practical Limitations

The findings of this study should be interpreted in light of several practical limitations. First, when an infant is partially covered by clothing or bedding, respiration-related motion may still be captured through optical flow if the resulting surface displacement remains sufficiently visible in the video. However, under severe occlusion, such as when thick, heavy, or tightly constrained bedding suppresses both appearance-based changes and respiration-induced surface motion, the visual respiratory signal may become substantially weakened or unavailable. Second, although the grid-based analysis and Z-score normalization used in the proposed framework may reduce direct dependence on infant body size or apparent body scale, the effects of variations in body size were not independently quantified in the present study. Similarly, the camera-to-infant distance was not systematically controlled or stratified in the AIR dataset, and therefore a validated operating range cannot be established. At greater distances, subtle respiratory displacements may be represented by fewer pixels and become more difficult to detect, whereas excessively close camera placement may limit full-body coverage and reduce the spatial area available for adaptive respiratory-region localization. Third, the proposed framework was designed to reduce sensitivity to illumination changes through the use of YCrCb-based color information, Z-score normalization, and the combined analysis of color/intensity differences and optical flow. Nevertheless, severe or rapidly varying illumination changes, particularly when they introduce temporal components within the respiratory frequency range, may still affect the extracted respiratory information. Likewise, although the framework evaluates periodicity within the predefined respiratory frequency band of 0.2–0.8 Hz to reduce the influence of transient or irregular body movements, non-respiratory motion that repeatedly occurs within the same frequency range cannot be completely excluded. Additionally, the primary objective of this study is to adaptively localize respiration-related regions and estimate respiratory rate across different sleep postures, rather than to directly detect or classify abnormal respiratory events such as apnea. The current framework therefore does not include a dedicated mechanism for detecting apnea events or determining the absence of a valid respiratory signal when respiratory periodicity is substantially reduced or temporarily absent. Finally, although subject-wise cross-validation was employed, the present evaluation included only 15 infants; therefore, the findings should be interpreted as a proof-of-concept validation, and broader generalizability remains to be established using larger and more heterogeneous infant cohorts. Future studies should validate the proposed framework using datasets that encompass a wider range of body sizes, camera-to-infant distances, illumination conditions, occlusion levels, and non-respiratory movements, as well as datasets containing clearly annotated apnea events.

## 7. Conclusions

This study proposed an analytical approach for identifying valid respiratory regions across the entire video frame without relying on predefined anatomical ROIs or pretrained object detection models, taking into account that respiration-related information may appear in different body regions depending on an infant’s sleep posture. Temporal color/intensity variations and optical flow-derived motion changes were extracted from RGB and IR videos, and the frequency characteristics of these signals were combined to evaluate respiratory periodicity for each grid cell, thereby enabling adaptive selection of valid respiratory regions. Using the single anchor grid cell selected in this manner, respiratory rate estimation achieved an MAE of 1.9953 BPM in 6-fold subject-wise cross-validation, with MAEs below 2.5 BPM for the supine, side-lying, and prone postures. These results suggest that respiration-related information in infants may not be confined to predefined anatomical regions but may be manifested in different body regions depending on sleep posture. However, owing to the limited availability of public infant datasets, generalization performance could not be evaluated using a large external dataset. Further validation using datasets that include a wider range of subjects and sleep environments is therefore warranted.

## Figures and Tables

**Table 1:** Effect of the number of grid cells on respiratory rate estimation performance.

Grid Cell Count	MAE	RMSE	PCC
1	1.9953	3.9681	0.7287
2	2.1927	4.515	0.6666
3	2.2536	4.6323	0.6638
5	2.1716	4.4131	0.6734
Whole Grid Cells	5.2820	8.0419	0.2238

Note. Performance was evaluated using a single grid cell as well as aggregations of the top 2, 3, and 5 grid cells.

**Table 2:** Performance comparison of ROI strategies for respiratory rate estimation.

ROI Type	N	MAE	RMSE	PCC
Chest	327	4.1893	6.8649	0.4094
Body	327	4.0394	6.8765	0.4111
Torso	327	6.0633	9.0863	−0.0194
Anchor Grid Cell	327	2.1836	4.2289	0.7216

Note. The Anchor Grid Cell represents the adaptive ROI selection strategy proposed in this study. Chest, Body, and Torso ROIs were obtained using the pretrained YOLOv8 model provided for the dataset, and the detected bounding boxes were cropped and used as predefined anatomical ROIs. The same respiratory signal extraction and respiratory rate estimation procedures were applied to all ROI configurations. Videos for which a valid YOLOv8-based ROI could not be obtained were excluded from this comparison. To ensure a fair comparison, all ROI strategies, including the proposed Anchor Grid Cell approach, were evaluated using the same subset of 327 videos. Accordingly, the Anchor Grid Cell performance reported in this table represents the result obtained from this common N = 327 subset and differs from the primary result obtained using the complete 374-video dataset. All reported performance metrics were calculated from the held-out test predictions aggregated across the 6-fold subject-wise out-of-fold (OOF) evaluation. MAE denotes Mean Absolute Error, RMSE denotes Root Mean Square Error, and PCC denotes Pearson Correlation Coefficient.

**Table 3:** Posture-specific respiration rate estimation performance using 6-fold subject-wise cross-validation.

Posture	Ratio	MAE	RMSE	PCC	SR_3BPM	SR_5BPM
Supine	60.20%	1.9823	4.1492	0.5252	85.33%	88.44%
Side	32.10%	2.1508	3.929	0.8423	81.67%	87.50%
Prone	7.80%	1.4531	2.3697	0.3071	89.66%	96.55%

Note. The table presents posture-specific performance derived from the out-of-fold (OOF) predictions of the 6-fold subject-wise cross-validation conducted on 15 infants. In each fold, data from 12–13 infants were used for training and validation to optimize the model weights and thresholds, whereas data from the remaining 2–3 infants were reserved exclusively for testing and were not involved in parameter tuning. The OOF predictions from all 374 test videos across the six folds were aggregated and categorized according to sleep posture: supine (n = 225, 60.2%), side-lying (n = 120, 32.1%), and prone (n = 29, 7.8%). MAE denotes mean absolute error, RMSE denotes root mean square error, and PCC denotes Pearson correlation coefficient. SR denotes success rate and is defined as the proportion of videos with an absolute error between the estimated and reference respiration rates within 3 BPM (SR_3BPM) or 5 BPM (SR_5BPM), respectively.

**Table 4:** Validation performance across correlation coefficient thresholds and weight combinations between the two modalities for parameter selection.

Threshold	Fusion (Flow:Color)	Avg_ Selected_Cells	MAE	RMSE	PCC	ICC
0.5	0.6:0.4	6.59	2.3058	4.6874	0.6504	0.6445
	0.7:0.3	6.59	2.2237	4.5647	0.6609	0.6562
	0.8:0.2	6.59	2.3256	4.7711	0.6382	0.6313
	0.9:0.1	6.59	2.1796	4.4517	0.6733	0.6694
0.6	0.6:0.4	5.65	2.4119	4.8416	0.6301	0.6228
	0.7:0.3	5.65	2.238	4.5075	0.6638	0.6601
	0.8:0.2	5.65	2.2796	4.5706	0.6578	0.6532
	0.9:0.1	5.65	2.2268	4.5153	0.6646	0.6599
0.7	0.6:0.4	4.6	2.3184	4.7149	0.6453	0.6389
	0.7:0.3	4.6	2.2212	4.4815	0.6676	0.6633
	0.8:0.2	4.6	2.2286	4.4823	0.6701	0.6655
	0.9:0.1	4.6	2.2042	4.4508	0.6741	0.669
0.8	0.6:0.4	3.21	2.4073	4.8984	0.6276	0.6171
	0.7:0.3	3.21	2.3533	4.806	0.6304	0.6225
	0.8:0.2	3.21	2.1751	4.3833	0.6886	0.6831
	0.9:0.1	3.21	2.1069	4.2279	0.7057	0.7014
0.9	0.6:0.4	1.81	2.3784	4.8116	0.6549	0.6383
	0.7:0.3	1.81	2.2055	4.5188	0.6819	0.6707
	0.8:0.2	1.81	2.0392	4.2208	0.7225	0.7129
	0.9:0.1	1.81	2.0119	4.1216	0.7332	0.7251

Note. Threshold refers to the correlation coefficient criterion applied to adjacent grid cells within a 3 × 3 neighborhood of the Anchor Grid Cell. Fused (Flow:Color) indicates the relative weights assigned to the optical-flow and color-difference features. Avg_Selected_Cells represents the average number of grid cells selected for respiration estimation among the training/validation videos used for parameter selection. The parameter sweep in Table 4 was conducted specifically for the optional Chain Extension configuration and was not required for the primary single-Anchor Grid Cell configuration. Parameter selection was performed exclusively on the training/validation portion of each fold, and the corresponding held-out test subjects were not used during this process. MAE denotes Mean Absolute Error, RMSE denotes Root Mean Square Error, PCC denotes Pearson Correlation Coefficient, and ICC denotes Intraclass Correlation Coefficient.

**Table 5:** OOF Performance Comparison of Baseline Methods and the Proposed Method.

Model	ROI	MAE	RMSE	PCC
EfficientPhys	Body ROI	4.034	6.928	0.377
AIRFlowNet	Body ROI	3.926	7.070	0.445
Optical Flow		3.4107	6.2998	0.3726
Color Differences		4.2538	6.5312	0.2834
**Anchor Grid Cell**		**1.9953**	**3.9681**	**0.7287**
Anchor Grid Cell + Chain Extension		2.1510	4.3701	0.6714

Note. This table compares the performance of existing non-contact respiratory rate estimation methods with that of the proposed framework under the same 6-fold subject-wise out-of-fold (OOF) evaluation protocol. The performance metrics for EfficientPhys and AIRFlowNet were obtained by reimplementing the models on the dataset used in this study based on Song et al. (2025) [16]. These models estimate respiration from body regions of interest (ROIs) localized using a pretrained YOLOv8 model. The proposed framework comprises two configurations: Anchor Grid Cell, which estimates respiratory information using a single selected grid cell, and Anchor Grid Cell + Chain Extension, which additionally incorporates neighboring cells satisfying the correlation criterion within a predefined spatial range. The results reported in this table were calculated by aggregating the predictions obtained for the held-out test subjects across all six folds. MAE denotes Mean Absolute Error, RMSE denotes Root Mean Square Error, and PCC denotes Pearson Correlation Coefficient. Bold values indicate the best performance for each metric among the compared methods.

**Table 6:** Results of 6-fold subject-wise cross-validation based on out-of-fold (OOF) test data.

Fold	Fusion (Flow:Color)	MAE	RMSE	PCC	ICC	SR_3BPM
1	0.9:0.1	2.4093	4.5753	0.4167	0.3456	85.00%
2	0.9:0.1	1.6344	3.4161	0.6394	0.5505	89.39%
3	0.9:0.1	2.5344	5.0402	0.5058	0.3511	78.57%
4	0.9:0.1	1.3188	2.2614	0.9615	0.9562	90.00%
5	0.9:0.1	2.8989	5.1719	0.1321	0.1075	70.37%
6	0.9:0.1	1.2616	2.2819	0.8525	0.8478	92.19%

Note. This table presents the out-of-fold (OOF) test results of the 6-fold subject-wise cross-validation conducted on 15 infants. In each fold, data from 12–13 infants were used for training and validation to optimize the fusion weights and correlation threshold, while data from the remaining 2–3 infants were reserved as test data and were not used for parameter tuning. Fused (Flow:Color) denotes the fusion weight ratio assigned to Optical Flow and Color Differences, respectively. MAE, mean absolute error; RMSE, root mean square error; PCC, Pearson correlation coefficient; ICC, intraclass correlation coefficient. SR denotes Success Rate and is calculated as the proportion of videos for which the absolute error between the estimated and reference respiration rates is within 3 BPM or 5 BPM, respectively.

**Table 7:** Comparison of signal-combination strategies for respiratory rate estimation.

Method	MAE	RMSE	PCC
**Spectral Multiplication (Ours)**	**1.9953**	**3.9681**	**0.7287**
Weight Average	2.3496	4.4496	0.6266
Optical Flow Only	2.4057	4.6727	0.6485
Color-Intensity Differences Only	4.2665	7.3681	0.3466

Note. Spectral multiplication denotes the proposed frequency-domain combination, in which the magnitude spectra of the optical-flow and color/intensity signals are multiplied, i.e., (|FFT(F)| × |FFT(C)|). Weighted average (5:5) denotes an equal-weight combination of the two modalities in the time domain followed by Fourier transformation, i.e., (|FFT(0.5F + 0.5C)|). Flow only and Color only denote the use of (|FFT(F)|) and (|FFT(C)|), respectively. For all four cases, the same preprocessing, breathing-frequency range, periodicity scoring criterion, Top-1 Anchor Grid Cell selection rule, and subsequent respiratory-rate estimation procedure were retained; only the strategy used to construct the periodicity representation for anchor localization was varied. Bold values indicate the best performance for each metric among the compared methods.

**Table 8:** Robustness to global and localized non-respiratory periodic interference.

Interference Amplitude	Global MAE (BPM)	Local MAE (BPM)	Local SR_3BPM	Anchor Escape
0.1	2.244	2.096	83.2%	5.1%
0.2	3.530	2.833	79.4%	8.6%
0.3	4.602	3.760	73.5%	12.8%
0.4	5.823	4.941	64.4%	20.1%
0.5	7.021	5.772	57.8%	48.9%

Note. Non-respiratory periodic interference was simulated by injecting a 0.6-Hz sinusoidal component within the respiratory frequency band (0.2–0.8 Hz). Interference amplitude was defined relative to the in-band signal magnitude of each grid cell. Global contamination was applied to all 576 grid cells, whereas localized contamination was restricted to a 6 × 6 region centered on the originally selected Anchor Grid Cell. Anchor escape denotes the proportion of clips in which the newly selected anchor moved outside the contaminated local region. SR_3_ represents the proportion of respiratory-rate estimates with an absolute error within 3 BPM.

## Data Availability

Publicly available datasets were analyzed in this study. The AIR-125 dataset can be found at https://github.com/ostadabbas/Infant-Respiration-Estimation?tab=readme-ov-file (accessed on 19 June 2026), and the AIR-400 dataset can be found at https://github.com/michaelwwan/air-400 (accessed on 19 June 2026).

## Abbreviations

The following abbreviations are used in this manuscript:

BPM	Breaths per minute
Cb	Blue-difference
Cr	Red-difference
CV	Cross-validation
FFT	Fast Fourier transform
GT	Ground-truth
ICC	Intraclass correlation coefficient
IR	Infrared
MAE	Mean absolute error
OOF	Out-of-fold
PCC	Pearson correlation coefficient
RMSE	Root mean square error
ROI	Region of interest
SD	Standard deviation
SIDS	Sudden infant death syndrome
SR	Success rate
